# Programmatic Challenges in Managing Multidrug-Resistant Tuberculosis in Malawi

**DOI:** 10.4103/ijmy.ijmy_47_21

**Published:** 2021

**Authors:** Loveness Charlie, Bibie Saidi, Emnet Getachew, Cathreen Lydiah Wanjiru, Mekdelawit Abebe, Hanna Amanuel Tesfahunei, Mary Gorret Atim, Tsegahun Manyazewal, Ronald Nachipo Mlera

**Affiliations:** 1 Center for Innovative Drug Development and Therapeutic Trials for Africa, College of Health Sciences, Addis Ababa University,; 2 KNCV TB Foundation, Challenge TB Project, Blantyre, Malawi,; 3 Kibong’oto National Tuberculosis Hospital, Kilimanjaro, Tanzania,; 4 Department of Public Health, College of Health Science, Arsi University, Asella, Ethiopia,; 5 Department of Nursing, Saint Peter Tuberculosis Specialized Hospital, Addis Ababa,; 6 Hager Biomedical Research Institute, Asmara, Eritrea,; 7 Department of Clinical Pharmacy, Soroti Regional Referral Hospital, Soroti, Uganda,; 8 Department of Professional Practice and Conduct, Nurses and Midwives Council of Malawi, Lilongwe, Malawi

**Keywords:** Extensively drug-resistant tuberculosis, Malawi, multidrug-resistant tuberculosis, programmatic management

## Abstract

**Background:**

Multidrug-resistant tuberculosis (MDR-TB) is one of the most urgent challenges that Malawi tends to take a firm public health action. A recent increase in multidrug MDR-TB cases, a decrease in treatment success rate, and a double increase of lost-to-follow-up call into question the country’s programmatic management of MDR-TB (PMDT). As such, the study aimed at exploring programmatic challenges in managing MDR-TB in Malawi.

**Methods:**

A comprehensive and nonsystematic search was made in PubMed and Google Scholar using mainly the keywords “MDR-TB” “extensively drug-resistant TB,” Malawi. The study reviewed existing guidelines and gray literature and reviewed data obtained from the national TB program (NTP) as well.

**Results:**

The study found the following challenges affecting PMDT: decrease in funding, partial access to GeneXpert, delay in diagnosis, long treatment duration, lack of adequate personal protective equipment, the long turnaround time of culture results, failure to initiate all diagnosed patients on treatment, absence of alternative second-line medicines, and lack of transport from health facilities to patient homes.

**Conclusion:**

If the Malawi NTP is to achieve a vision of a “TB-free Malawi,” rigorous efforts at all levels must be made, including mobilizing domestic resources for improved MDR-TB program performance. Developing partners should continue providing the much-needed funding to the Malawi government to stand in the wake of the MDR-TB crisis.

## Introduction

Multidrug-resistant tuberculosis (MDR-TB) threatens global TB control efforts and it is one of the greatest challenges facing public health, particularly in resource-poor settings where adequate diagnosis and treatment are often unavailable.^[1,2]^ MDR-TB is described as TB resistant to at least isoniazid and rifampicin, two of the most important anti-TB drugs. The continuing spread of drug-resistant-TB (DR-TB) is one of the most urgent and difficult challenges facing global TB control. According to the World Health Organization (WHO) 2020 annual TB report, an estimated 10 million people fell ill with TB in 2019 and close to half a million developed rifampicin-resistant TB, of which 78% had MDR-TB.^[3]^ The extent and burden of MDR-TB vary significantly from country to country and region to region. Malawi is one of the top 28 high-burden TB/human immunodeficiency virus (HIV) countries in the world with a 46% coinfection rate, and MDR-TB is an emerging issue. Based on the Malawi national TB program (NTP) data from 2017 to 2018, the country has experienced a double increase in MDR TB cases. In 2018 alone, 126 laboratory-confirmed cases of MDR-TB were reported, of which 107 started treatment from 58 cases in 2017.^[4]^

To ensure effective management of DR-TB, Malawi introduced several activities to be implemented as a part of core program strategies. In line with WHO’s END TB strategy by 2035 (3), the activities involve prevention, case detection, care and treatment, surveillance, drug management, and monitoring and evaluation of program performance.^[5–8]^ The Malawi NTP coordinates these activities and is referred to collectively as the “programmatic management of drug-resistant TB” (PMDT). The PMDT central team works hand in hand with MDR-TB district teams in implementing its activities across the country.

Diagnosing, treating, and caring for a person affected with MDR-TB pose enormous managerial challenges in any health-care system even for those in high-income settings. Globally, the implementation of PMDT is facing challenges such as diagnosis and treatment of MDR TB where probable MDR TB patients are being missed (only 51% of people with bacteriologically confirmed TB were tested for rifampicin resistance [RR] in 2018) and for those diagnosed, not all were initiated on treatment where only one in three of the approximately half a million people who developed MDR/RR-TB in 2018 were treated.^[9,10]^ Poor funding to NTPs was also contributed to the further spread of the disease in several countries; dependence on treatment regimens that are complex and toxic and coinfection with HIV that leads to poor treatment outcomes.^[11–14]^

Malawi, being a resource-constrained country, is also facing similar challenges evidenced in the burden of TB and MDR-TB.^[15–17]^ An increase in MDR-TB cases and poor treatment outcomes presents a formidable challenge to TB control that will lead to failure in achieving the end TB strategy. Therefore, it is the main objective of this paper to explore the programmatic challenges that Malawi NTP is facing in managing MDR-TB. Specifically, the study focused on challenges regarding case detection, care and treatment, prevention, drug management, surveillance, and financing MDR-TB.

## Methods

A comprehensive and nonsystematic search was made in PubMed and Google Scholar using mainly the keywords “MDR-TB,” “extensively drug-resistant TB,” Malawi. The existing national guidelines and gray literature were also reviewed to identify additional relevant information. The study also used data obtained from the NTP database.

## Results

### The extent of multidrug-resistant tuberculosis in Malawi

**Figure 1:**
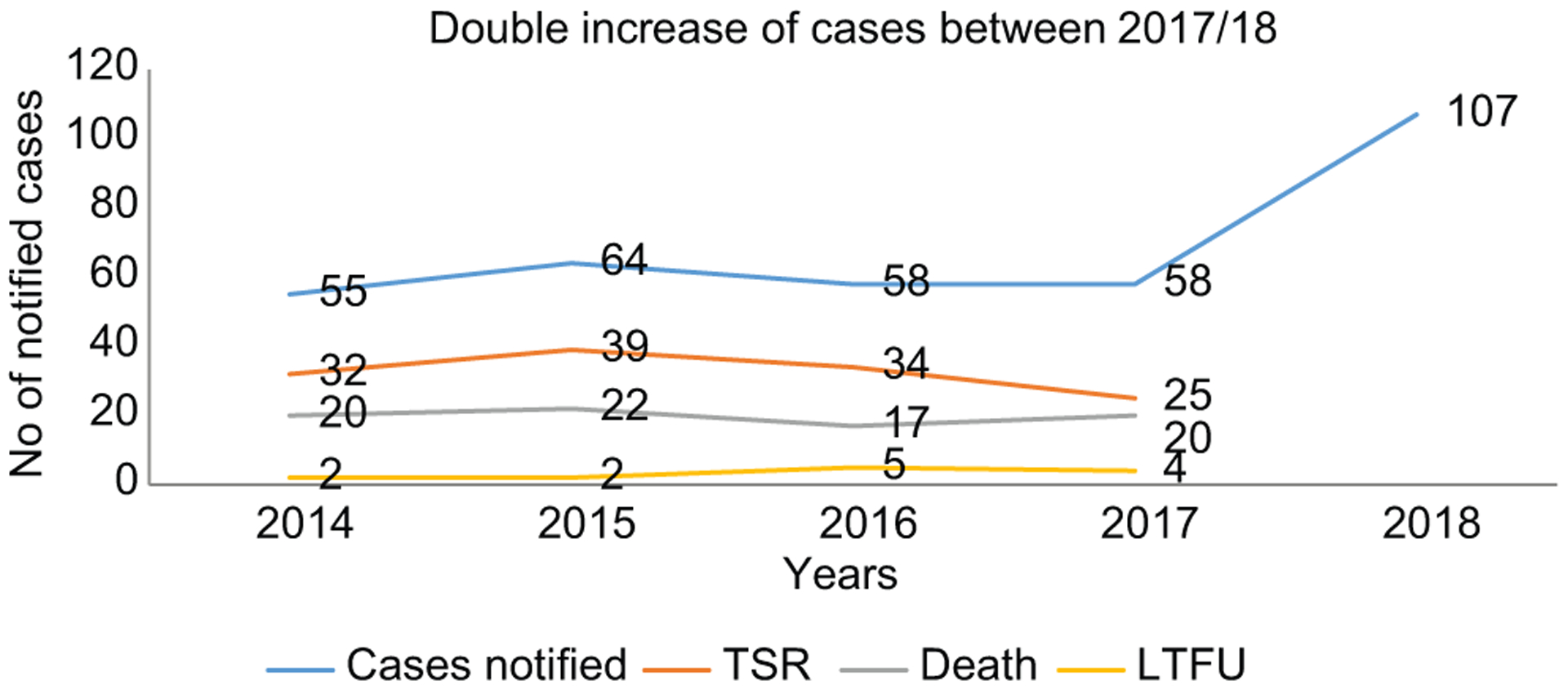
Trends of multidrug-resistant tuberculosis cases and outcomes in Malawi, 2017–2018

Malawi reported an estimated proportion of 6.4% MDR/RR-TB among previously treated cases and 0.75% new cases, while MDR/RR-TB incidence was at 2.3/100,000 population in 2018.^[10]^ In 2011 through a nationwide drug resistance survey, the MDR-TB prevalence rate was 0.4% among new smear-positive TB patients and 4.8% among retreatment patients.^[18]^ There was a double increase in cases that were initiated on treatment between 2017 and 2018 as shown in Figure 1.

### Programmatic management

#### Case detection

NTP introduced systematic TB screening in all health facilities in the country. With systematic TB screening, all clients/patients attending the health facility regardless of health condition are screened for TB.^[19]^ Once a TB presumptive is identified, they are requested to submit sputum samples for laboratory examination. The study found that microscopy (both light and florescent) remains the mainstay for TB diagnosis in Malawi, with radiology as an adjunctive technology was available.^[19,20]^ The NTP also recommended GeneXpert MTB/RIF as the initial screening tool for DR-TB at the health facility level in nationally prioritized patients groups. Thus, the sputum sample submitted is either examined on microscopy or Xpert machine depending on eligibility criteria.

**Figure 2:**
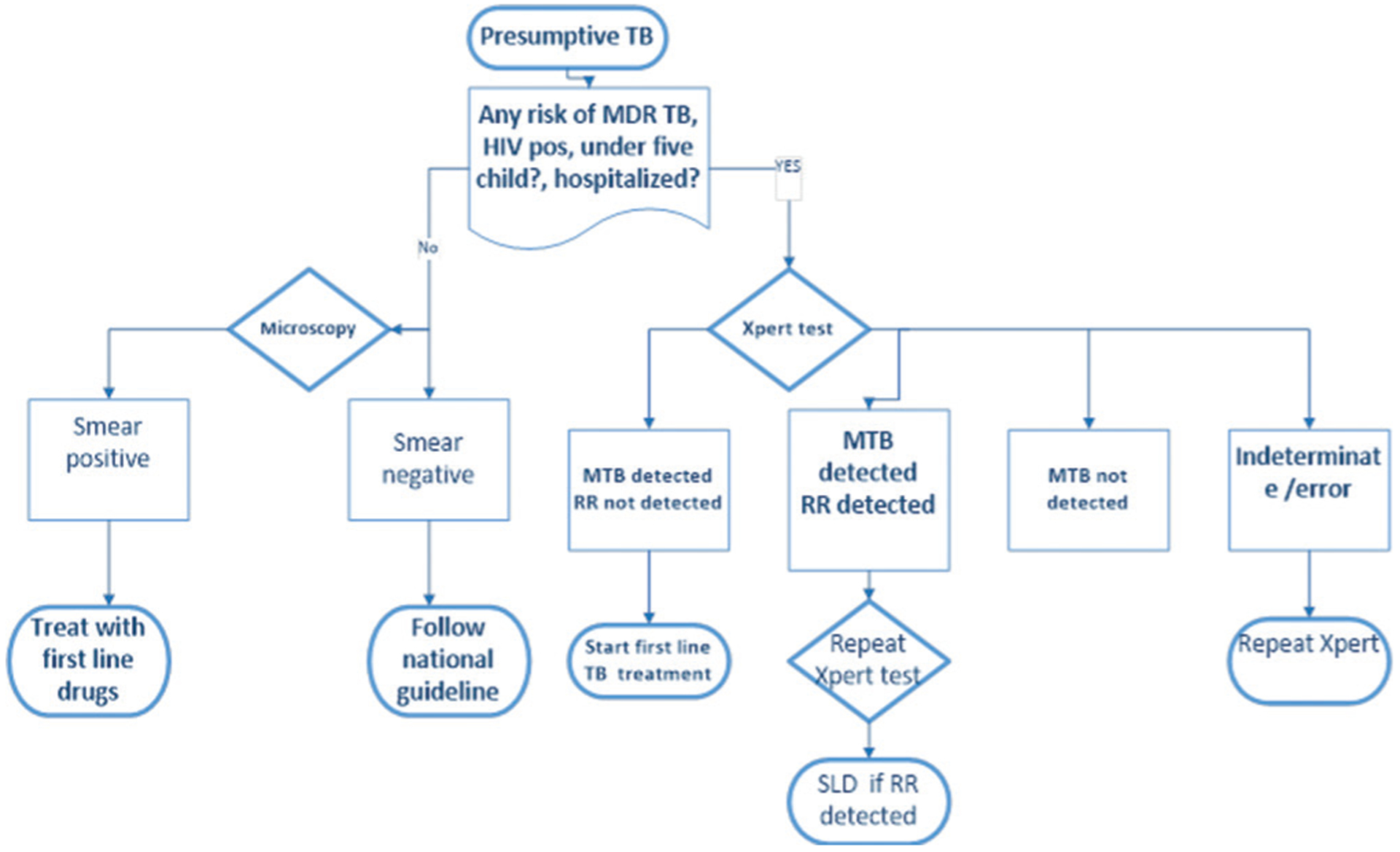
National algorithm for diagnosing tuberculosis in Malawi

The Xpert algorithm dictates the test of sputum samples of only those that are presumed to be at risk for MDR-TB and the rest of the samples are examined on microscopy (light-emitting diode or fluorescent). The genotypic drug sensitivity test (DST) is selectively used on hospitalized patients, health-care workers (HCWs), contacts of MDR patients, people living with HIV (PLHIV), previously treated patients, and patients failing on first-line drugs because they are considered as at-risk groups for MDR TB. If sputum results from Xpert examination are resistance positive, the Xpert test is repeated as a confirmatory test, and when the repeated sample remains positive, another sputum sample is taken and sent to National TB Reference Laboratory (NTRL) for culture, while the patient is started on the second-line drugs (SLDs).^[20,21]^ If the second confirmatory Xpert test is negative, the patient is started on first-line anti-TB treatment. The study further identified that the country has one NTRL and two regional cultural laboratories that provide high-level diagnostic services including phenotypic testing in liquid and solid media to confirm TB and drug sensitivity testing on selected specimens from across the country. Results turnaround time from the NTRL depends on how much workload is available and culture media (solid or liquid) used. Solid culture media takes 21–42 days for growth, while liquid media take 5–10 days, and culture is considered as a gold standard for diagnosing MDR TB. Figure 2 shows the national algorithm for diagnosing TB in Malawi.

#### Care and treatment

Malawi manages MDR-TB cases using both shorter and conventional (longer) regimens with a total treatment duration of 18–20 months (based on conversion) for the latter. When there is no complicated DR pattern a shorter regimen of 9–12-month (4–6 months with amikacin – moxifloxacin – Ethionamide [prothio-manide] – Clofazimine – Pyrazinamide – high dose isoniazid/ethambutol/5 months with moxifloxacin–clofazimine–pyrazinamide-ethambutol) is used.^[22]^ While longer regimen of 8 Cm-Lfx-Cs-Eto-Z/12 Lfx-Cs-Eto-Z is used for complicated cases such as pregnant women and those with extrapulmonary MDR-TB.^[23,24]^ The cases are being managed under a community- and outpatient-based strategy where patients are receiving treatment from health workers/treatment supporters in their homes and undergo longitudinal clinical follow-up at zonal DR-TB Referral Clinics once a month.^[18]^ Zonal clinics involve clinical examination such as the collection of follow-up monthly sputum for microscopy and culture tests for confirmed RR-/MDR/TB patients enrolled in second-line treatment. The study also identified that not all MDR TB diagnosed patients are traced and put on treatment for instance in 2018 only 107 cases were put on treatment out of 126 diagnosed. On treatment outcomes, NTP data for the past years indicated treatment success rate is decreasing by 22%, a 50% increase of lost to follow up, and a slight decrease of death by 2%.^[26]^ Through consultation with one TB officer, the study has also found that, in the course of treatment, not all HWCs are willing to provide care and support to MDR TB patients. HCWs deliberately shun away from their duties for fear of being infected when they come in contact with MDR TB patients.

#### Prevention

Malawi has been tackling TB Infection Prevention and Control (IPC), in general, using various aspects and training of HCWs has been the main focus. NTP together with its partners has been training HCWs of different cadres on TB IPC with much emphasis on the importance of implementing administrative and environmental control measures as well as the use of personal protection equipment (PPE)^[18]^ (masks for infectious patients and respirators to protect HCWs and visitors from potential infections) in their respective facilities. Due to limited funding, the NTP is failing to provide enough PPEs to facilities with priority being given to TB officers.

The NTP with support from partners such as the Royal Netherlands TB Foundation (KNCV) with funding from USAID (2015 to 2019) implemented the Challenge TB project.^[19]^ Among other things, the project implemented activities of TB IPC through active case finding and Finding cases Actively Separating safely and Effective treatment (FAST). The strategies involve triaging, isolation, fast-tracking, and installation of direction fans in all crowded areas at a facility, diagnosis (provider-initiated asking TB cardinal questions and safe sputum collection and examination), and treatment. With the purpose to inactivate TB bacilli in suspended droplet nuclei, the NTP also installed germicidal ultraviolet parts in specific patient care areas such as MDR TB wards, X-ray units, and large waiting areas/rooms.^[21,25]^ The country is also providing isoniazid as a preventive treatment for latent TB infection to under-five children who are a household contact of pulmonary TB cases and PLHIV.^[19]^

#### Drug management

The central medical store system allows ordering of drugs of MDR TB when a patient is diagnosed unlike with drug-susceptible TB where ordering is done quarterly. The study found that it takes at least 1 week for the request/order to be processed, while the patient is waiting to be initiated on treatment.

### Multidrug-resistant tuberculosis surveillance

Malawi is implementing systematic TB screening in all patients that come to the facility including PLHIV.^[20]^ Those with one or more TB symptoms (presumptive) are asked to submit sputum and those diagnosed are tracked by health surveillance assistants to be initiated on treatment.

#### Tuberculosis financing

The study found that annual funding for TB activities has been decreasing over the years with 62% remain unfunded in 2019.^[10]^

## Discussion

The situation surrounding RR/MDR-TB has worsened in recent years. The situation is even more worrying if one considers that only 25% of these patients are receiving effective treatment, and among those that do, only 56% are achieving cure.^[10]^ In other words, only about 10% of cases of MDR-TB worldwide are being cured. Although recent decades have witnessed increased efforts in the fight to end TB, fundamental gaps are hampering these efforts, particularly in resource-constrained settings and in settings with a high burden of disease.

The main challenge in MDR TB management is the underfunding of NTPs.^[21,28]^ Over the years, the funding to Malawi NTP has been decreasing and currently, 62% of existing TB activities remain unfunded.^[18]^ If the global MDR-TB epidemic is to be controlled, at least 90% of patients must be detected and offered treatment, and 90% must be cured.^[29]^ To improve detection and to reduce the time to diagnosis, all suspected TB cases must be tested with rapid molecular detection, using GeneXpert or another similar technique.^[13]^ However, due to limited funding, the country is failing to implement universal access to genexpert which is a molecular method that detects rifampicin-resistant within 2 h. This partial access to the Xpert examination has resulted in missing cases and late diagnosis as other samples are examined using microscopy which cannot detect resistance. In 2019, the WHO reported that not all DR-TB cases are diagnosed-only 51% of people with bacteriologically confirmed TB were tested for RR in 2018 and not all DR-TB cases were treated (only one in three of the approximately half a million people who developed MDR/RR-TB in 2018 were treated). The delay in diagnosis has led to a further spread of disease and poor treatment outcomes.^[1,21]^ The latest treatment outcome data for people with MDR/RR-TB show a global treatment success rate TSR of 56% with other MDR-TB burden countries such as Bangladesh, Ethiopia, Kazakhstan, and Myanmar going beyond with a better TSR of >70%. While in Malawi, TSR has been decreasing by 22% between 2014 and 2018. To further aggravate the problem, the currently recommended therapeutic regimens for DR-TB are complex, expensive, and have poor efficacy and tolerability.^[10,12]^

MDR-TB reduces responses to standard short-course chemotherapy with first-line anti-TB drugs, leads to higher mortality and treatment failure rates, and increases the period of transmissibility of the disease, resulting in super-resistant strains that have emerged.^[1]^ Malawi TB NSP 2015–2020 highlighted that the absence of alternative second-line medicines (SLDs) to replace failing regimens/drugs is contributing to failure in achieving a desirable TSR.^[21,22]^ In addition, the long treatment duration has also resulted in other patients default treatment and also compromise adherence, especially in continuous phase resulting to further spread of disease as well as poor treatment outcomes.

Culture-based phenotypic DST methods are currently the gold standard for DR-TB detection, but these methods are time consuming and require sophisticated and well-established laboratory infrastructure, qualified staff, and strict quality and infection control.^[8]^ The high workload and few technicians to process the samples at NTRL have led to the long turnaround time of culture results which makes them difficult to use for confirming and evaluating treatment of DR patients.^[21]^

Over the years, the program has been facing transport challenges to take the health workers to and from patients’ homes which result in patients often missing daily injections hence compromising adherence and risking treatment failure. Lack of transport has also resulted in no regular checkup and clinical assessment of MDR TB cases through outreach arrangements further contributing to treatment failure as patients are not frequently monitored for side effects that may lead to changing of regimen or some drugs within the regimen. During the monthly zonal clinic’s patients use public transport to and from the facility even without a mask that might result in further disease transmission.

Failure to trace all diagnosed patients and put them on treatment has also further contributed to the increase of cases as these missed patients continue to spread the disease. For instance, in 2017, 126 cases were diagnosed, but only 107 were put on treatment. In addition, delay in instituting diagnosed and traced MDR TB patients on treatment due to the central medical store’s drug ordering process has also contributed to the spread of disease as patients wait for quite a period to be initiated on drugs hence the increase in cases.

The absence of isolation facilities for the admission of complicated MDR TB cases poses a big problem to the management of complicated MDR cases. HCWs tend to improvise a room within the TB ward hence risking further spread of disease to both HCWs and patients.

The stigma associated with the disease has also led to the further spread of disease and compromise the care to be given to MDR TB patients. With stigma, patients opt not to wear face masks or they wear if they hear an HCW’s motorbike or vehicle while other patients fail to disclose their disease to family members or the community. Some patients deliberately withdrew from family activities, based on erroneous beliefs about disease transmission, to protect loved ones and family member’s control practices which have led to a lack of support for them. Other HCWs also deliberately deny providing care and support in fear of being exposed to the disease.

Despite preventive measures being critical for reducing the burden of TB disease among individuals exposed, however, Malawi is providing isoniazid to under five susceptible TB contacts and PLHIV. MDR TB contacts who do not fit the above category are just assessed for active TB. If they are asymptomatic, they are sent back home and advised to report back to the facility if they experience any symptoms. Due to limited funding, the program has also been struggling to provide enough personal protective equipment to the HCWs and patients with priority being given to TB officers which has contributed to the increase. Failure of the NTP to sustain strategies introduced by implementing partners is also another factor that has contributed to poor management of MDR TB cases. It has been a trend was if partners complete their project everything that was being done also ceases regardless of being helpful.

## Conclusion

If the Malawi NTP is to achieve a vision of a “TB-free Malawi,” rigorous efforts at all levels must be made including mobilizing domestic resources for improved MDR-TB program performance. Developing partners should continue providing the much-needed funding to the Malawi government to stand in the wake of the MDR-TB crisis. All stakeholders should continue tracking (surveillance) of local epidemics of DR-TB to minimize transmission; NTP should ensure that there is good collaboration between central and district teams to ensure effective management of MDR-TB patients and resources.
